# “The molecule’s the thing:” the promise of molecular modeling and dynamic simulations in aiding the prioritization and interpretation of genomic testing results

**DOI:** 10.12688/f1000research.8600.3

**Published:** 2016-07-25

**Authors:** Gavin R. Oliver, Michael T. Zimmermann, Eric W. Klee, Raul A. Urrutia

**Affiliations:** 1 Division of Biomedical Statistics and Informatics, Department of Health Sciences Research, Mayo Clinic, Rochester, MN, 55905, USA; 2 Center for Individualized Medicine, Mayo Clinic, Rochester, MN, 55905, USA; 3 Laboratory of Epigenetics and Chromatin Dynamics, Epigenomics Translational Program, Gastroenterology Research Unit, Departments of Biochemistry and Molecular Biology, Biophysics, and Medicine, Mayo Clinic, Rochester, MN, 55905, USA

**Keywords:** Individualized Medicine, Clinical Genomics, Diagnostic Odyssey, Oncology, Molecular Modeling, Molecular Dynamics, Variants of Unknown Significance

## Abstract

Clinical genomics is now a reality and lies at the heart of individualized medicine efforts. The success of these approaches is evidenced by the increasing volume of publications that report causal links between genomic variants and disease. In spite of early success, clinical genomics currently faces significant challenges in establishing the relevance of the majority of variants identified by next generation sequencing tests. Indeed, the majority of mutations identified are harbored by proteins whose functions remain elusive. Herein we describe the current scenario in genomic testing and in particular the burden of variants of uncertain significance (VUSs). We highlight a role for molecular modeling and molecular dynamic simulations as tools that can significantly increase the yield of information to aid in the evaluation of pathogenicity. Though the application of these methodologies to the interpretation of variants identified by genomic testing is not yet widespread, we predict that an increase in their use will significantly benefit the mission of clinical genomics for individualized medicine.

In under a decade, sequencing of a human genome moved from a three billion dollar, multi institutional effort, to a common research assay. Now, 16 years after completion of the draft sequence, genomic assays are an increasingly prevalent component of clinical testing. Oncology, hematology and the diagnosis of rare, Mendelian genetic disorders (diagnostic odyssey) have particularly benefited from the increased genetic testing resolution afforded by modern sequencing technologies. The commercialization of clinical assays to profile patients’ somatic or germline genomes is creating the potential for higher resolution diagnoses and individualized treatment options. Stories of the resounding success of such efforts have been widely and justifiably publicized, and have now captured imaginations well beyond the laboratory or clinic; even so far as the White House and US Congress. The President’s Precision Medicine Initiative ^1^ has now been signed into existence and guarantees expanded exploration within this burgeoning area of health sciences research.

While sequencing technologies enable the interrogation of entire genomic, transcriptomic and epigenomic repertoires, current clinical implementations remain heavily biased toward the protein coding component of the DNA, either in the form of whole-exome sequencing or gene panel testing. This is a reflection of both the relative maturity of the field of molecular genetics and the clinical familiarity with both it and the concept of molecularly targeted therapy, which currently represents the major paradigm of tailored cancer treatment. Limiting clinical interrogation of a patient genome to a coding subset may seem counterintuitive in the era of whole-genome sequencing, but the reality is that our understanding of non-coding genomic variation and its role in disease remains in its infancy, particularly with regard to clinical actionability. The explosion of genomics research has increased our understanding of the human genome and the wide-ranging functions it encodes, however most regions of the genome remain uncharacterized and poorly understood. Even with genomic testing limited to protein coding regions, the majority of sequence variants detected go unreported or are classified as variants of uncertain significance (VUSs) due to their uncertain impact on normal physiological function.

For the researchers and clinicians on the ground of this nascent field, enthusiasm is high but it is tempered with frustration due to the as-yet high rate of cases for which genomic testing remains insufficiently informative (around 75% for diagnostic odyssey) ^2, 3^.

A recurring scene is the convening of large, multidisciplinary teams of clinical and research staff to pore over lengthy lists of genomic sequence variants, exchange professional opinion and debate next steps. However, in the end, these involved efforts often fail to identify variants with highly confident causal or mechanistic relationships with the disease phenotype. In oncology, the knowledge gained for treatment selection is often compelling, but with little prior evidence, most are difficult to actualize. For example, identification of a novel missense mutation in the functional domain of a known, druggable oncogene might logically appear to be a therapeutic target, but no information may exist linking the mutation to drug efficacy. It is this disconnect between novel genotypes identified and their link to the patient’s phenotypes which thwarts our ability to further improve clinical decision making. Thus, there is a significant need to overcome this critical challenge to expand the success of genomic testing in individualized medicine.

The most mature applications of clinical exome and panel testing are the detection of focal substitutions (missense, nonsense or splice site), insertions and deletions (INDELS), or copy number changes. While copy number gains or losses can intuitively be interpreted as potential gain-of-function or loss-of function events respectively, point mutations and INDELS are less easily characterized in terms of their effects on protein function. Wet-lab assays designed to ascertain functional relevance of specific mutations are a highly desired solution, but they remain cumbersome, time-consuming and cost prohibitive in the great majority of cases. They are therefore misaligned with the high throughput nature of modern genomics. Meanwhile, computational methods of predicting the pathogenicity of genomic sequence alterations exist and are both amenable to high-throughput predictions and widely applied in the field, to the extent that they form a component of the American College of Medical Genetics and Genomics (ACMG) guidelines for the clinical interpretation of sequence variants ^4^. These algorithms utilize varied information including evolutionary conservation, genomic position or basic protein-level structural information to assign probabilistic scores or categorical predictions of pathogenicity. Their accuracy varies widely, with alternative tools often producing conflicting predictions for the same variant. Furthermore, the predictions often lack contextualization in terms of biological effect. Algorithmic categorization of a variant as pathogenic offers little insight into the nature of its phenotypic effect and less indication still of whether it is relevant clinically or how to act upon it. Many researchers continue to work to improve these methods, but the burden of VUS interpretation persists and the need for a means to address this burden and facilitate clinical interpretation is widely felt within the field.

The aforementioned computational tools largely leverage genome-centric information. While there is much proven value to these data, the biological reality comprises many additional layers of complexity. Mutations affect atomic-level biophysical changes that may alter the structure and biochemical function of the genome's protein products. While we detect variants in DNA, we typically interpret their effect by inferring or confirming their deleterious effect on encoded proteins ( Figure 1). Even the effects of regulatory variants are typically interpreted as altering the probability of a protein to be expressed or spliced into a given functional form. Thus ideally, any method used to assess pathogenicity of genomic variants should possess the ability to look beyond genomic annotation to a functional context, at an atomic resolution. This increased resolution is likely to yield information that can add context to mutations, better identify the mechanism of pathogenicity, and combine with existing knowledge to facilitate in clinical decision making.

**Figure 1.**
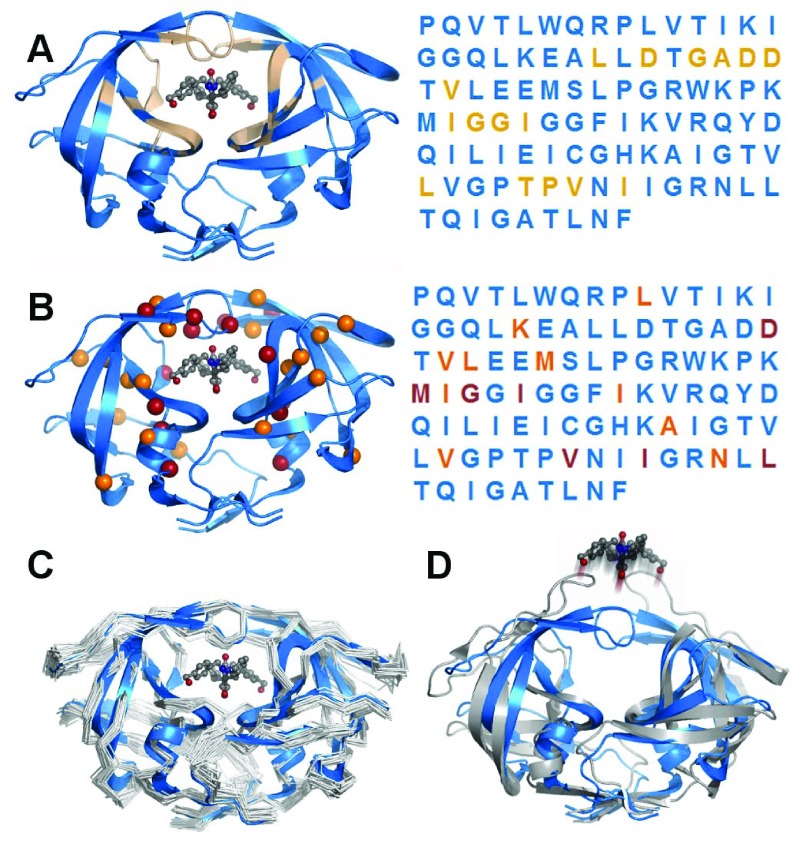
The HIV-1 protease as a generic model system for computational biophysics ^14– 16^. HIV-1 protease has become a model system because of its disease relevance, the availability of mutational and drug binding data, and for its tractable size and molecular stability. The protein’s function is to cleave HIV peptides into the functional proteins of the infectious HIV virion. This example illustrates generalized concepts of the applicability of protein modeling methods in clinical investigations **A**) *Ligand binding residues are spatially separated.* The functional protein dimer is shown with a pharmacologic inhibitor bound to the active site. Residues that are within 3.5Å of the inhibitor are highlighted in tan. The primary sequence is colored identically to the three-dimensional structure to indicate relative positioning of residues. It is apparent that the ligand binding portion of the protease consists of residues that are non-adjacent within the primary sequence, which illustrates an advantage of modelling over linear sequence analysis. **B**) *Drug resistance mutations tend to occur in residues within the active site.* Many mutations have been characterized that are associated with resistance to inhibitor drugs. While the sites of these mutations are also disjoint in sequence, nearly all of them fall into the same set of drug binding residues, indicating how modeling can enable prediction of a mutation’s effect. **C**) *Structural effects of mutations beyond the active site.* Drug-resistance mutations in non-ligand-binding residues have been shown, using computational experiments, to impact the flexibility of the protein and therefore alter drug binding. Computational modeling has characterized the flexibility of the protein in multiple mutated states, illustrating the potential to predict the functional effect of mutations beyond an active site. **D**) *Dynamics of ligand-free protein*. Computational studies have the advantage of being able to simulate conditions that are difficult to assay experimentally, such as the dynamics of ligand-free forms in atomic detail.

Computational biophysics and biochemistry aim to understand molecular function in a dynamic manner at an atomic resolution differing from their wet-lab counterparts methodologically but sharing the same goals. The most obvious advantages of computational approaches include the potential to test hypotheses *in silico* that would be difficult, costly, or intractable in the lab. Similar to laboratory experiments, computational calculations and simulations are most interpretable when they are well designed, test a specific hypothesis, contain positive and negative controls, connect the data generated to the pre-existent knowledge in the field, and allow drawing further functional inferences. When all these conditions are met, the three dimensional and dynamic representation of the modeled mutations may add a significant value to the interpretation of a genomic test’s finding.

Motivated by the conditions described, we have begun to apply molecular protein modeling and dynamic simulation techniques in the interpretation of genomic variants identified by next generation sequencing. These methodologies abandon the practice of regarding mutations as occurring in linear strings of nucleotides or amino acids and instead offer a three dimensional, dynamic view, at an atomic resolution. Furthermore they are inherently compatible with the current state of clinical genomics testing and its predominant focus on point mutations within protein coding regions. The methods involve the computational generation, optimization and verification of a protein structural model, often based on homology to an experimentally determined protein structure ^5, 6^. *Ab initio* modeling is also possible, albeit with lower confidence. The model itself contains information about the linear amino acid sequence of the protein, along with the relative spatial coordinates of its atoms. Precise mathematical and biophysical parameters in the form of a force field are applied to the model to calculate energetic characteristics of the system. The methods are often mature and under reasonable conditions can be expected to produce a model with error comparable to that of a typical structure solved by nuclear magnetic resonance spectroscopy.

Molecular modeling allows us to visualize the manner in which proteins are folded to create a functional structure and to accurately simulate how this is disrupted by mutational events. Because we can visualize atomic bonds, mutations which disrupt *inter*-molecular interactions - for example between an enzyme and its substrate - can also be modeled. To illustrate their function, many enzymes are analogized to traditional tools. One example likens proteases and scissors; if a variant inhibits the closing motion of the scissors about their fulcrum, the blades cannot function; if a variant blocks entry of a material between the blades, then that material can no longer be cut. The reality, of course, is more complex. Proteins are flexible polymers that only achieve mechanistic accuracy by folding into complex and specific three dimensional conformations that restrain or focus thermal fluctuations towards collective motions that are typically part of the mechanism itself. Regions of the properly folded structure’s surface that interact with other molecules, either for chemical modification (e.g. phosphorylation) or structural contacts (e.g. α/β tubulin assembly), can also be critical for function and modified by variants. In addition to the native shape of a protein, the ability of the linear amino acid polymer to achieve that shape is critical. Protein folding often occurs through progressive assembly of local structural elements or intermediates. If an intermediate is either stabilized or destabilized by a variant, the ability of the protein to achieve the native fold could be altered. 

Of course, molecular modeling methods have limitations. The generation of a reliable model is often dependent on the pre-existence of experimentally determined homologous structures and even where these exist, they may provide only partial information ^7^. A model is by nature an approximation of reality. Nonetheless, current techniques have achieved suitable accuracies such that they are frequently used ^8– 11^ in applications including drug design, virtual screening, protein engineering and site-directed mutagenesis. With this in mind, their relatively slow uptake in the clinical setting is somewhat surprising. This fact may simply reflect the relative nascence of clinical genomics and the tendency of specialists to seek out fields in which their specialty is already known and accepted.

We encourage those working within or in proximity to the clinical genomics setting to engage in or promote increased exploration of these methods in their work. Our initial experiences of applying such techniques at the clinical-research boundary of genomics-driven oncology, hematology and diagnostic odyssey have been encouraging and have begun to inform decision-making. We are observing clear initial benefits in regard to variant prioritization, interpretation and validation, with several initial publications in preparation to highlight the value obtained. Of course, not everyone will possess the necessary scientific knowledge or technical skills to deploy these methods directly, but we propose that inter or intra-institutional collaborations may enable those lacking the requisite expertise to identify and access appropriate resources. Alternatively, several freely available online solutions exist ^12, 13^ and enable a researcher or clinician to experiment with core modeling methods in the absence of extensive technical expertise. Such independent or collaborative exploration will open the door to deeper understanding of biological mutations and has the potential to inform clinical thinking. Wider use of these methods will enable the field to progress toward standardization of methodologies and approaches, and aid in the formulation of clinical best-practices that will serve to strengthen initiatives like the ACMG standards.

As we alluded to earlier in this article, wider ‘omics’ research is a broad and varied landscape of many alternative techniques that are continually revealing new findings with potential diagnostic and therapeutic relevance. Applications such as transcriptomics and epigenomics are beginning to develop clinical presence, but currently they lag behind DNA-based methods. Within the realm of DNA sequencing, the potential clinical relevance of non-coding variation is recognized and bioinformatics developments as well as major experimental initiatives like The Encyclopedia of DNA Elements (ENCODE) bring us closer to routine exploitation of such findings in the clinic. Multi-omic and systems-level research are also widening the scope in which we consider the molecular underpinnings of disease and these promise to have productive futures in clinical settings. Furthermore, novel treatments including oligonucleotide-based and immunogenic therapies are emerging, and move beyond the current prevailing targeted therapeutic paradigms. The case we have made for increased use of protein modeling focuses intentionally on the current state of clinical genomics testing and related diagnosis and treatment decision-making, rather than within these burgeoning research areas. Our opinion is that protein modeling satisfies a current and real need within clinical genomics and that its application should be immediately explored and encouraged. This is not to say it’s clinical niche will be short-lived as many emerging techniques may well benefit from similar modeling methods being applied in either central or supportive roles, for example in nucleic acid structural modeling or in predicting neo-antigen affinities for MHC-class molecules.

In summary, genomic testing is assuming an increasingly prominent role in the clinical identification, prioritization, and interpretation of disease-associated genomic variants. While a few of these variants are known to be pathogenic, knowledge of the deleterious effects of the majority remain elusive. Laboratory methods remain gold-standards for functional characterization, but are generally incompatible with large-scale characterization of variant effects. Predictive algorithms are in some instances successfully applied to differentiate pathogenic variants from variants of uncertain significance, however these methods largely ignore measures of protein structure, energies, molecular bonds, intermolecular interactions, post-translational modification effects, protein aggregation, and stability. Conversely, this information lies at the heart of molecular modeling and dynamic simulation, which collectively equip us more fully to grapple with interpreting the effects of VUSs. We strongly believe that these methodologies can be major components in forging the analytical pipeline for interpreting genomic testing in individualized medicine and advocate their increased deployment within variant characterization efforts.
