# Supplementary material for: “The molecule’s the thing:” the promise of molecular modeling and dynamic simulations in aiding the prioritization and interpretation of genomic testing results
Source: F1000Res. 2016 Jul 25;5:766. Originally published 2016 Apr 27. [Version 3] doi: 10.12688/f1000research.8600.3 (PMC4920209; doi:10.12688/f1000research.8600.3)
Supplement: Peer review history

# Peer review: Referee response for version 2

**DOI:** 10.5256/f1000research.9676.r14497

**Published:** 2016-06-24

**Authors:** Saverio Alberti

**Affiliations:** 1 Unit of Cancer Pathology, Center of Excellence for Aging Sciences and Translational Medicine (CeSI-MeT), "G. d'Annunzio" University of Chieti-Pescara, Chieti, Italy

A clearer and more pointed presentation of key points is provided.

 A broader discussion may begin on issues that remain open, such as large-scale crystallization efforts/ 'universal' protein modeling or on the role of mutations in non-coding DNA.

 This may well form the basis for additional articles on this topic

I have read this submission. I believe that I have an appropriate level of expertise to confirm that it is of an acceptable scientific standard.

# Peer review: Referee response for version 2

**DOI:** 10.5256/f1000research.9676.r14496

**Published:** 2016-06-23

**Authors:** Attila Bérces

**Affiliations:** 1 Omixon, Inc., Budapest, Hungary

This revision significantly improved from the original article. Neoantigen prediction is a good example, where atomic level modelling can significantly contribute to clinical decisions.

I have read this submission. I believe that I have an appropriate level of expertise to confirm that it is of an acceptable scientific standard.

# Peer review: Referee response for version 1

**DOI:** 10.5256/f1000research.9254.r13603

**Published:** 2016-05-31

**Authors:** Anita Grigoriadis

**Affiliations:** 1 Breast Cancer Now Unit, Cancer Division, Faculty of Life Sciences & Medicine, King's College London, London, UK

In this article Oliver *et al. *highlight to importance of analytical methods such as “molecular modeling and molecular dynamic simulations" as tools that can significantly increase the interpretation of the functional impact of mutations/polymorphisms currently classified as variants of unknown significance (VUSs).

 While the topic of molecular dynamics simulation for VUSs is worth discussing, the article could expand on

- the current limitation of these methods
- the complexity of these nucleotide changes in e.g. coding and non-coding genomic regions
- integration with other high throughput technologies and thus increase challenges for multimodal effects in such simulations
- HIV is given as an example, however other clinical examples, their associated specificity and limitations should be provided to make this article relevant for a wider audience.

I have read this submission. I believe that I have an appropriate level of expertise to confirm that it is of an acceptable scientific standard, however I have significant reservations, as outlined above.

# Peer review: Referee response for version 1

**DOI:** 10.5256/f1000research.9254.r13880

**Published:** 2016-05-18

**Authors:** Attila Bérces

**Affiliations:** 1 Omixon, Inc., Budapest, Hungary

The authors of this article "highlight a role for molecular modeling and molecular dynamic simulations as tools that can significantly increase the yield of information to aid in the evaluation of pathogenicity."

 Molecular dynamics simulations are indeed excellent tools to study protein and nucleic acid structures, protein-ligand, protein-protein and protein-nucleic acid interactions. These structural changes and interactions are fundamental in molecular biology and thus molecular simulations are an excellent tool to increase our understanding of these biological processes. In particular the proposed method may help the understanding of coding variants associated with amino acid substitution. In addition, RNA structure can be modeled, which can be informative about some micro-RNA variants.

 However, these methods are not without limitations and this paper does not state these limitations explicitly. Pathogenic effects come in all strength and complexities. Most often pathogenicity is not associated with a single variant, but with a combination of several variants and the effect of a particular variant  depends on the genotypes of other genes. Most likely, variants will stay of unknown significance unless we develop a system level understanding of biology. We may reach a point that we can simulate viral functions at molecular level, but it will not be applied to human variants any time soon.

 Molecular modelling has some limitations and requires expert knowledge to apply it properly.

 While I agree that molecular modelling is an excellent tool to understand molecular biology, we need to develop a system level of understanding of the connection between molecular biology and genetics before we can apply molecular simulations. Currently, molecular modeling can make practical contribution to understand variants associated with strong effects or the molecular basis of some Mendelian disorders.

I have read this submission. I believe that I have an appropriate level of expertise to confirm that it is of an acceptable scientific standard, however I have significant reservations, as outlined above.

# Peer review: Referee response for version 1

**DOI:** 10.5256/f1000research.9254.r13600

**Published:** 2016-05-06

**Authors:** Saverio Alberti

**Affiliations:** 1 Unit of Cancer Pathology, Center of Excellence for Aging Sciences and Translational Medicine (CeSI-MeT), "G. d'Annunzio" University of Chieti-Pescara, Chieti, Italy

In this article the Authors seek ways on how to better interpret the functional impact of mutations/polymorphisms currently classified as variants of unknown significance (VUSs). To tackle such a problem, the authors propose “molecular modeling and molecular dynamic simulations as tools that can significantly increase the yield of information to aid in the evaluation of pathogenicity.” Expectations are that this “will significantly benefit the mission of clinical genomics for individualized medicine.”

The idea is good and deserves to be published. Most of all, it deserves to be extensively discussed.

Points to be discussed

As much as the idea is good, it would benefit from better highlighting its (current) limitations.

 

1. *Ab initio* molecular modeling is a difficult enterprise. It can rarely be performed to some extent of accuracy by automatized engines/softwares and often reaches good structure prediction in around 60% of cases.
  Several large-scale, tentatively whole-coding genome, protein crystallization initiatives have been undertaken. Discussion of the state of the art of these initiatives may provide metrics on what could be expected from real-life application of the Authors’ proposal, and how this is expected to evolve in the near future.
2. Most mutations/polymorphisms are detected in non-coding regions of DNA. This is hardly surprising given that non-coding DNA represents approximately 97% of the total content of a mammalian cell. The authors mention mutations in non coding regions, but this deserves being more extensively discussed in the publication. 

Suggestions

Discussion of the points above and of the suggestions below, may possibly lead to extend the proposed approach to additional layers of prediction.

Algorithms are there that do recognise promoter, enhancer and super-enhancer regions with increasing accuracy. Clearly, allocating a mutation/polymorphism in one such region would increase its investigational and possibly medical value.

Additional regions of interest are CpG islands upstream or, less reliably, within coding regions.

Additional regions of interest are anchoring sites of DNA loops to distinct nuclear/nuclear membrane regions.

Additional regions of interest are those hosting the increasing family of non-coding RNA (NCRNA), including long NCRNA and miRNA, that have been shown to play distinct regulatory functions and may have roles in cancer development.

I have read this submission. I believe that I have an appropriate level of expertise to confirm that it is of an acceptable scientific standard, however I have significant reservations, as outlined above.
